# A 64-year-old woman with rapid neurologic decline diagnosed with *Toxoplasma* encephalitis after presumed metastatic cancer

**DOI:** 10.1177/2050313X211019784

**Published:** 2021-05-24

**Authors:** Claudia Rosso, Lisa Giscombe, Toufic Tannous, Matthew Keating

**Affiliations:** 1 University of California, Irvine School of Medicine, Irvine, CA, USA; 2 Mercy Hospital Medical Partners, Miami, FL, USA; 3 Division of Hematology and Oncology, Mercy Hospital, Miami, FL, USA; 4 Roger Williams Medical Center, Providence, RI, USA; 5 Boston University School of Medicine, Boston, MA, USA; 6 Division of Hematology and Oncology, University of California, Irvine, Irvine, CA, USA

**Keywords:** Toxoplasmosis, HIV, anemia, metastatic, ring-enhancing lesion

## Abstract

In an era of fragmented medical care, concurrent clinical features that ultimately lead to a unified diagnosis may not be prioritized appropriately. We present a case of a 64-year-old woman referred to hematology clinic for anemia, with recent memory loss and gait disturbance. Two months later, she developed pneumonia; imaging workup showed a left renal mass. Neurologic function continued to decline precluding definitive nephrectomy. She then presented with new-onset seizure and initial neuro-imaging was reported as unremarkable. One month later, outpatient neurologic workup demonstrated new left-sided weakness which prompted hospitalization and repeat neuro-imaging, which showed a 1.7-cm right frontal lobe mass lesion with surrounding vasogenic edema. The patient ultimately underwent craniotomy with resection of the mass lesion; pathology did not show metastatic renal cell cancer, the provisional clinical diagnosis. Rather, immunostaining revealed a parasite and ultimately led to a diagnosis of *Toxoplasma* encephalitis, an infection whose clinical presentation had been interpreted by healthcare providers for months to be a result of metastatic cancer.

## Introduction

Toxoplasmosis is a parasitic infection that often lies dormant in individuals with a healthy immune system. Contracted by ingestion of contaminated food (pork or shellfish), water, soil, or cat feces, the parasite may compromise the brain, eyes, or other organs.^1^ Immunocompromised patients are most at risk, particularly in untreated HIV/AIDS patients. In these patients, cerebral toxoplasmosis accounts for nearly 90% of toxoplasmosis cases versus extracerebral involvement.^2^ When possible, clinical diagnosis of cerebral toxoplasmosis via exam, imaging, and serologies is preferable to biopsy given the risks associated with the invasive biopsy procedure. The presence of multiple, ring-enhancing lesions on magnetic resonance imaging (MRI) lends further credence to the diagnosis. Despite being a parasitic infection, fever is not present in approximately half of cases. Patients present with seizures, focal neurological deficits, and a wide spectrum of mental status changes.

Both toxoplasmosis and primary central nervous system lymphoma should be considered in an HIV-positive patient presenting with brain lesions. In the event of de novo HIV/AIDS and toxoplasmosis, careful attention should be paid to the sequencing of and response to treatment. Timing of antiretroviral therapy for newly diagnosed HIV/AIDS patients with pre-existing toxoplasmosis infections is controversial given the risk of immune reconstitution syndrome. However, limited studies do support initiation of antiretroviral therapy within the first 2 weeks after starting toxoplasmosis therapy.^3^ We present a case of a 64-year-old woman referred to hematology clinic for anemia, who experienced rapid neurologic decline and was ultimately diagnosed with HIV-associated *Toxoplasma* encephalitis after presumed metastatic cancer.

## Case presentation

A 64-year-old Haitian woman who moved to the United States 3 years ago, with past medical history of esophageal candidiasis, hypertension, and normocytic anemia of unclear etiology with unremarkable esophagogastroduodenoscopy and colonoscopy 2 years ago, presented to the hematology clinic of our hospital for workup of her anemia. Comprehensive lab workup was sent (Table 1). Iron studies suggested anemia of chronic disease, and an incidental monoclonal gammopathy of undetermined significance (MGUS) was detected. She had a small paraprotein spike of 0.31 g/dL on serum protein electrophoresis on the initial clinic visit with negative serum immunofixation, creatinine 1.23 mg/dL (0.5–1.1 mg/dL), and hemoglobin 8.9 mg/dL (11.8–16 mg/dL). However, her clinical symptoms on initial presentation to hematology clinic were predominantly neurological: memory loss, cognitive delay, gait impairment, falls, and urinary incontinence. Formal neurological evaluation and Human Immunodeficiency Virus (HIV) testing were recommended via the hematology consult note to her primary care office, but unfortunately these recommendations were not done due to fragmentation of care. The hematologist did not follow through to see that the recommendations were carried out expediently, and the primary care physician did not see the recommendations in a timely manner.

**Table 1.** Laboratory results from anemia workup on initial clinic visit and 3 months later, respectively.

	Initial clinic visit	Three months later
Hemoglobin (11.8–16 mg/dL)	8.9	9.5
Mean corpuscular volume (80–97 fL)	91	93
Platelets (150–400 K/uL)	191	162
White blood cells (4–11 K/uL)	7.3	4.8
Creatinine (0.5–1.1 mg/dL)	1.23	1.33
Calcium (8–10.5 mg/dL)	10.3	10.2
Total protein (6.8–8.6 g/dL)	9.4	9.1
Albumin (3.5–5 g/dL)	4.3	4
Iron (51–146 mcg/dL)	51	86
Total iron binding capacity (240–450 mcg/dL)	227	225
Ferritin (10–109 ng/mL)	N/A	1061
Serum immunofixation	No monoclonal immunoglobulin detected	N/A
Serum protein electrophoresis	Monoclonal peak detected measuring 0.31 g/dL	N/A
Free light chain ratio (0.26–1.65)	1.19	N/A
Methylmalonic acid (87–318 nmol/L)	363	285
Folate (5.8–31.4 ng/mL)	17.3	N/A
Vitamin B12 (213–816 pg/mL)	439	N/A
TSH (0.35–4.9 uIU/mL)	0.64	N/A

N/A: not available.

TSH: Thyroid-stimulating hormone.

The patient was hospitalized 2 months later at an outside hospital for pneumonia, at which time a Computerized Tomography (CT) scan revealed an incidental 5.7 cm mass in the upper pole of her left kidney, suspicious for malignancy. There were tentative plans for laparoscopic left nephrectomy depending on optimization of her nutritional and functional status. She then presented to the emergency department of a third hospital 1 month later with a new-onset seizure of undetermined etiology. CT and MRI of the brain were reportedly negative for any acute findings. Electroencephalogram (EEG) was abnormal and she was started on levetiracetam. She followed up in hematology clinic for repeat lab work to monitor her anemia and MGUS (Table 1). But again, her neurological symptoms superseded her blood dyscrasias: she had worsening gait impairment requiring full assistance and appeared lethargic with minimal verbal responses. Once more, the hematologist reiterated the urgent need for neurological evaluation. One month later, outpatient clinical exam by a neurologist revealed left upper extremity apraxia, myoclonus, and confusion, prompting inpatient admission. Further clinical exams in the hospital detected left arm weakness and left-sided hyperreflexia. The patient was nonverbal and did not follow commands. History was significant for progressive decline over the past several months, including presentation of memory loss, markedly reduced speech with terse and delayed responses, impaired gait with multiple falls, intermittent urinary incontinence, over 30 pounds of weight loss with marked decline in activities of daily living, and bed-bound status for most of the day.

During her inpatient stay, EEG showed triphasic waves and slowing but no epileptiform changes. Contrast MRI of the brain showed a right frontal lobe enhancing lesion measuring up to 1.7 cm with surrounding vasogenic edema and mass effect with 3 mm leftward midline shift, overall concerning for a metastatic process. Non-contrast MRI of the cervical spine was completed as the patient was unable to tolerate a contrast study; findings included heterogeneous appearance of the bone marrow and degenerative changes. She was treated with intravenous dexamethasone and radiation oncology and neurosurgery were consulted for the brain lesion. Radiation oncology advised deferring radiation treatment in favor of definitive diagnosis and resection. Neurosurgery performed craniotomy and resection of the right frontal lesion.

CT scan of the chest was performed for staging purposes as the leading suspicion remained a metastatic process, with renal cancer as the presumed primary. The CT scan showed an incidental 3.3-cm nodule in the right thyroid gland, extending into the upper mediastinum. Thyroid studies were consistent with euthyroid sick syndrome, and per endocrinology consultation the thyroid mass was unlikely to give rise to distant metastases in the absence of locoregional findings. Therefore, the thyroid mass was deferred for non-urgent, outpatient workup. Ultimately, per pathology review, the frontal lobe lesion was consistent with acute on chronic inflammation with necrotic tissue concerning for infection rather than malignancy.

Numerous *Toxoplasma* gondii organisms were identified on immunostaining, and *Toxoplasma* IgG was positive. Thus, the patient was diagnosed with *Toxoplasma* encephalitis. She began treatment with Sulfadiazine, Pyrimethamine, and Leucovorin. Subsequently, she tested positive for HIV in the setting of Acquired Immunodeficiency Syndrome (AIDS)-defining illness, with absolute CD4 count of 30/mm^3^, and HIV-1 RNA quantitative polymerase chain reaction (PCR) of 148,000 copies/mL. Initiation of antiretroviral therapy for HIV/AIDS was placed on hold while she continued treatment for her opportunistic infection, that is, *Toxoplasma* encephalitis. Her neurologic decline, beginning several months prior to hospitalization, was felt to represent a multifactorial etiology from HIV encephalitis and *Toxoplasma* encephalitis. She failed to show neurological improvement during her hospitalization, even requiring artificial nutrition via a nasogastric tube for a couple weeks postoperatively. She was felt by the primary team and multiple specialists to have very poor prognosis with little chance of meaningful neurological recovery, essentially mute and bed-bound with maximum assistance with all activities of daily living. Palliative care was consulted and comfort care has been implemented.

## Discussion

This case illustrates the pitfalls of fragmented medical care, and the temptation to focus on abnormal laboratory results (such as anemia) or imaging rather than the overall clinical picture, which was dominated by functional decline out of proportion to the degree of anemia. The patient also had known history of MGUS, but this did not explain her larger, more acute process. In MGUS patients, the risk of progression to multiple myeloma or other plasma-cell or lymphoid disorders is approximately 10% over a 10-year period.^4^ Plasmacytomas located in the kidneys or brain are exceedingly rare with less than 30 reported cases of each, respectively.^5,6^ The initial presumptive diagnosis for her known renal mass and newfound brain lesion was metastatic renal cancer. During her hospitalization, another distractor emerged in an incidental 3.3-cm thyroid mass that appeared on imaging. It would be quite unusual for a thyroid cancer to present with brain or renal metastasis without showing locoregional metastasis as well. With assistance from endocrinology consultation, it was determined that this thyroid mass was a non-factor in her active disease process and its workup was appropriately deferred to the outpatient setting.

In retrospect, there were a few clues in the outpatient and inpatient settings that may have led to more expedient diagnosis and treatment. The patient’s prior hospitalization was for pneumonia, but perhaps this presentation may have represented a *Toxoplasma* pneumonitis instead. Review of outpatient clinic charting shows report of blurry vision on review of systems, which may have been due to *Toxoplasma* chorioretinitis. The presence of esophageal candidiasis and weight loss in her history should have triggered a broad investigation for an underlying immunocompromised state beyond just cancer. Consideration for HIV is not infrequently overlooked in older adults.^7^ HIV testing was recommended at the initial hematology consultation in hematology clinic several months before her inpatient hospitalization, but unfortunately due to fragmentation of care, testing was not addressed until after the diagnosis of toxoplasmosis. As the patient was followed in outpatient specialty clinics, it became clear that her progressive neurological problems superseded her other medical conditions and therefore neurological consultation was expedited. While in the hospital, she completed an initial contrast MRI study, and when the finer details of the report are reviewed, one can see mention of an irregular ring of enhancement. A broad differential should be considered for any patient who presents with a ring-enhancing brain lesion^8–14^ (Table 2). Toxoplasmosis commonly presents with ring-enhancing lesions, although there are typically multiple lesions present rather than one solitary mass. It was not until the mass was biopsied that the possibility of HIV infection was considered. Multiple brain lesions are typical with cerebral toxoplasmosis, although a finding of a solitary lesion does not rule out the disease. In AIDS patients with cerebral toxoplasmosis, CT scans show a solitary lesion in as much as 39% of patients, whereas MRIs show a solitary lesion in less than 20% of patients.^15–18^

**Table 2.** Broad differential diagnosis for a ring-enhancing brain lesion, adapted from Garg 2010 and per review of available published literature on PubMed. Our patient has HIV-associated toxoplasmosis.

Differential diagnosis for a ring-enhancing brain lesion (adapted from Garg^11^)
Bacterial
Listeriosis
Mycobacterium avium–intracellulare
Pyogenic abscess
Tuberculoma and tuberculous abscess
Fungal
Aspergillosis
Cryptococcosis
Rhodococcosis
Zygomycosis
Histoplasmosis
Coccidioidomycosis
Nocardiosis
Mucormycosis
Paracoccidioidomycosis
Actinomycosis
Parasitic
Toxoplasmosis (often HIV-associated)
Neurocysticercosis
Amebic brain abscess
Echinococcosis
Cerebral sparganosis
Trypanosomiasis (Chagas disease)
Neoplastic
Primary CNS lymphoma (B- and T-cell subtypes)
EVB-positive diffuse large B-cell lymphoma
Leukemia
Meningioma
Oligodendroglioma
Astrocytoma (pilocytic, diffuse, or anaplastic)
Glioblastoma
Metastases
Inflammatory and demyelinating
Sarcoidosis
Systemic lupus erythematosus
Multiple sclerosis
Progressive multifocal leukoencephalopathy
Behcet’s disease
Whipple’s disease
Acute necrotizing encephalopathy
Acute disseminated encephalomyelitis
Vascular
Primary intracerebral hemorrhage
Hemorrhagic infarction
Ischemic infarction
Embolic infarction

CNS: central nervous system; EBV: epstein-barr virus.

The complexities of this patient’s case extended beyond simply when and what should be diagnosed and treated, as the management of expectations for long-term outcome even with aggressive treatment played a key role. Coexisting HIV encephalopathy and *Toxoplasma* encephalitis meant that even if the *Toxoplasma* was treated for the standard 6 weeks of therapy, the patient would have been unlikely to have much meaningful neurologic recovery. Her EEG findings of triphasic waves point to a very poor long-term prognosis. It could be argued that treatment of *Toxoplasma* and then HIV may hold some promise for her recovery. However, with other comorbidities yet to be fully worked up, and progressive decline over the past 6 months, to the point that the patient is fully dependent on others for activities of daily living and clinical decision-making, it became clear that comfort care will improve the patient’s quality of life going forward. Palliative care served to ease the transition out of the hospital for the patient and her family.

## Conclusion

*Toxoplasma* encephalitis is a parasitic infection that primarily affects the central nervous system, particularly in immunocompromised patients. In a patient with presumed cancer, the characteristic ring-enhancing brain lesions of Toxoplasmosis may be mistaken for metastasis if the overall clinical picture is neglected, as occurred in this case. Our patient fell victim to fragmented care and thus did not receive a more plausible diagnosis until after her craniotomy. Although she initially presented with signs consistent with HIV-associated toxoplasmosis, including rapid neurologic decline and esophageal candidiasis, the possibilities of HIV and *Toxoplasma* encephalitis were overlooked.
